# Helicobacter pylori Resistance: Morphomolecular Analysis of Formalin-Fixed Paraffin-Embedded (FFPE) Biopsy Specimens From 374 Patients

**DOI:** 10.7759/cureus.112809

**Published:** 2026-07-16

**Authors:** Torsten Hansen, Mathias Diederichs, Norbert Arens, Helena Philipps, Martin Balsliemke, Hauke Heinzow, Erwin Rambusch, Stefan Burg, Katharina Kriegsmann, Mark Kriegsmann, Joerg B Kriegsmann

**Affiliations:** 1 Pathology, Medical Care Center for Histology, Cytology, and Molecular Diagnostics Trier GmbH, Trier, DEU; 2 Internal Medicine II, Verbundkrankenhaus Bernkastel/Wittlich, Wittlich, DEU; 3 Internal Medicine II, Krankenhaus der Barmherzigen Brüder Trier, Trier, DEU; 4 Internal Medicine I, Klinikum Mutterhaus der Borromäerinnen gGmbH, Trier, DEU; 5 Internal Medicine, Kreiskrankenhaus Saarburg, Saarburg, DEU; 6 Clinical Chemistry, Rhein-Main Laboratory Medicine Practice MVZ GbR, Frankfurt, DEU; 7 Pathology, Center for Histology, Cytology and Molecular Pathology, Wiesbaden, DEU

**Keywords:** atrophy, clarithromycin, ffpe, fluoroquinolones, histology, h. pylori, h. pylori density, molecular pathology, resistance

## Abstract

Background: Antibiotic resistance is the leading cause of *Helicobacter pylori* eradication failure worldwide. Molecular testing of formalin-fixed paraffin-embedded (FFPE) gastric biopsy specimens enables simultaneous histopathological evaluation and detection of resistance-associated mutations.

Objective: This study aimed to investigate associations between histopathological findings and molecularly detected antibiotic resistance in *H. pylori*-positive FFPE gastric biopsies.

Methods: We analyzed FFPE biopsy specimens of 374 patients with *H. pylori* gastritis (mean age, 53.4 years; 40.1% male (n = 150); 59.9% female (n = 224)) using a strip assay-based molecular test to detect *H. pylori* and mutations conferring resistance to clarithromycin (R-CLA), fluoroquinolones (R-FLU), and dual resistance (R-CLA×FLU). Histological parameters, according to the updated Sydney system, included gastritis, chronic inflammation (mild, moderate, and severe), neutrophilic activity (absent, mild, moderate, and severe), *H. pylori* density (mild, moderate, and severe), atrophy, and intestinal metaplasia. Associations with these parameters were assessed by binary logistic regression.

Results: We found 67.1% resistance-associated mutations, most of them with R-CLA (n = 163; 64.9%), followed by R-CLA×FLU (n = 52; 20.7%) and R-FLU (n = 36; 14.3%). Overall, gastritis activity was mild in 213 patients (57%), moderate in 118 (31.6%), and severe in 20 (5.3%); chronic gastritis was absent in 23 patients (6.1%), mild in 87 (23.3%), moderate in 224 (59.9%), and severe in 63 (16.8%). *H. pylori* density was mild in 168 patients (44.9%), moderate in 149 (39.8%), and severe in 57 (15.2%). Severe *H. pylori* density was significantly associated with antibiotic resistance overall (odds ratio, 2.26; 95% confidence interval, 1.02-5.01; p = 0.045). Increasing age was significantly associated with resistant infections (p = 0.009). Gastric atrophy showed a nonsignificant trend toward association with clarithromycin and fluoroquinolone resistance (p = 0.06 for R-FLU and p = 0.07 for R-CLA, respectively).

Conclusions: Morphomolecular analysis of FFPE gastric biopsies represents a practical approach for routine *H. pylori* resistance testing. High *H. pylori* density may indicate an increased likelihood of antibiotic resistance. Given increasing resistance rates worldwide, molecular resistance testing is suggested to support individualized eradication therapy and improved antimicrobial stewardship.

## Introduction

*H. pylori* is a widespread Gram-negative bacterium infecting up to one-half of the global population. Although infection is often asymptomatic, it can be associated with severe conditions such as chronic gastritis, peptic ulcer disease, nonulcer dyspepsia, adenocarcinoma, and mucosa-associated lymphoid tissue lymphoma [1-3]. Thus, *H. pylori* is classified as a Group 1 carcinogen according to the International Agency for Research on Cancer. Moreover, its eradication reduces the incidence of gastric cancer [4]. Current treatment regimens for *H. pylori* eradication combine two or three antibiotics with one acid-suppressive drug for two weeks, aiming for eradication rates above 80% [3,5].

Despite constant efforts to improve treatment strategies, *H. pylori* has increasingly acquired antibiotic resistance through mutations [6-11]. Antibiotic resistance is a major driver of eradication failure; therefore, multiple studies have shown that susceptibility-guided therapy is more effective than empiric treatment [12-14]. Current guidelines recommend resistance testing prior to antibiotic therapy whenever feasible, including before first-line treatment to support optimized antibiotic stewardship [5,11,15].

The reference standard for antibiotic susceptibility testing is phenotypical analysis (agar dilution testing) using cultures of the organisms. However, successful phenotypic testing requires appropriate biopsy handling, special transport conditions, and an experienced laboratory [15]. Even under ideal conditions, results are rarely available rapidly enough for immediate therapeutic decision-making. Molecular testing is therefore increasingly used to confirm the presence of an *H. pylori* infection and to identify alterations in microbial gene sequences that result in antimicrobial resistance [11,16,17]. In this line, the Maastricht VI/Florence consensus report advises molecular methods, especially real-time polymerase chain reaction (PCR), whole-genome sequencing, and digital PCR, for detecting mutations associated with resistance to clarithromycin (R-CLA), fluoroquinolones (R-FLU), tetracycline, and rifampicin [5]. In addition, molecular resistance can also be analyzed in gastric biopsies routinely processed as formalin-fixed paraffin-embedded (FFPE) tissue for microscopy [9,18-24].

In addition to mutations that lead to antibiotic resistance, it has been demonstrated that changes in gastric histology patterns influence the bioavailability of eradication regimens. Mucosal atrophy and the degree of inflammation have previously been reported as possible relevant factors in this context [21,22]. In the present study, we analyzed FFPE gastric biopsies with an established diagnosis of *H. pylori* gastritis to assess the relationship between histopathological parameters and mutational status. The primary goal was to determine whether histopathological parameters, as defined by the updated Sydney system, were associated with *H. pylori* resistance. In particular, we first compared histopathology and overall resistance, followed by a specific comparison with R-CLA, R-FLU, and dual resistance (R-CLAxFLU).

## Materials and methods

Study population and molecular analysis

We performed a monocentric retrospective study on FFPE tissue specimens of the gastric mucosa with a microscopically established diagnosis of *H. pylori* gastritis. Inclusion criteria for morphomolecular diagnosis were as follows: 1) complete histopathological diagnosis and classification of gastritis according to the updated Sydney system [24]; 2) histological tissue specimens available for the second microscopical assessment; 3) molecular detection of *H. pylori*; and 4) molecular analysis for antimicrobial susceptibility or resistance as described in the following. The exclusion criteria were as follows: 1) noncomplete or missing histopathological diagnosis; 2) missing histological tissue specimens for a second microscopical assessment; 3) failed molecular detection of *H. pylori*; and 4) failed molecular analysis for antimicrobial susceptibility or resistance, as described below.

We examined 968 patients (male: n = 335, mean age = 50.1 years; female: n = 633, mean age = 51.4 years) by molecular testing from January 2014 to December 2023, as previously described [24]. Of this cohort, 374 patients met the inclusion criteria for the morphomolecular analysis in the present study. DNA was extracted from FFPE tissue blocks using the Maxwell RSC FFPE Plus DNA kit (Promega, Walldorf, Germany) [24]. Molecular detection of *H. pylori* and resistance-associated mutations for clarithromycin and fluoroquinolones was performed using a strip assay (GenoType Helico DR kit, Hain Lifescience GmbH, Nehren, Germany) including DNA amplification and hybridization, as previously reported [18,24]. The assay detects the major 23S ribosomal RNA mutations conferring R-CLA (A2146G, A2146C, A2147G) and the clinically relevant gyrA mutations at codons 87 and 91 conferring R-FLU [24]. Detection of mutant bands was interpreted as R-CLA only and/or R-FLU only. Cases positive for mutations conferring resistance to both were classified as R-CLA×FLU. Cases showing both wild-type and mutant probe hybridization were classified as resistant, reflecting the presence of mixed bacterial populations according to the manufacturer's instructions. Hybridization to WT only was defined as the absence of resistance-conferring mutations for both antibiotics (i.e., susceptibility) [24]. In particular, the strip includes internal positive/negative controls and an *H. pylori* identification band [24].

Concerning the visual analog scale of the updated Sydney system [25], we evaluated the graded variables as previously recommended: gastritis activity (mild, moderate, severe), chronic inflammation (mild, moderate, severe), and *H. pylori* colonization (mild (+), moderate (++), severe (+++)). In addition, we followed the recommendations for operative link for gastritis assessment (OLGA) according to the severity of gastric atrophy and for operative link on gastric intestinal metaplasia assessment (OLGIM) according to intestinal metaplasia [26]; patients were stratified into mild atrophy and mild intestinal metaplasia (OLGA I-II and OLGIM I-II, respectively), and moderate-to-severe atrophic gastritis and intestinal metaplasia (OLGA III-IV and OLGIM III-IV, respectively), as previously described [21]. In this cohort, all cases included both antral and corpus biopsies; for the assessment in this study, the biopsies were retrospectively evaluated together. The specimens were handled by standard procedure requirements for histopathological analysis following the updated Sydney system [25], including hematoxylin and eosin stain as well as Alcian blue periodic acid-Schiff stain, and modified Methylene Blue stain. In doubtful cases, immunohistochemistry was performed using antibodies against H. pylori (clone SP48; ready-to-use; Roche Diagnostics, Tucson, AZ) on the Benchmark Ultra platform (Roche Diagnostics). Histological analysis was performed in a routine diagnostic setting by experienced gastrointestinal pathologists and was retrospectively reviewed by two separate investigators (TH, MD) who were blinded to the mutational status results.

Statistical analysis

To identify the factors associated with overall *H. pylori* resistance as measured by the above-described method, followed by R-CLA, R-FLU, and R-CLA×FLU, binary logistic regression was conducted to estimate odds ratios with 95% confidence intervals. Multivariable binary logistic regression models were calculated for the explorative identification of histopathologic risk factors. The histopathologic parameters of the updated Sydney system (inflammation activity, inflammation chronicity, *H. pylori* activity, atrophy, and intestinal metaplasia) were included in the model simultaneously as independent variables. These were categorized (mild, moderate, severe), with the lowest grade serving as the reference category. Initially, the presence of general antibiotic resistance was defined as the dependent variable (1 = resistant isolates, 0 = sensitive isolates). Age (in predefined groups) and sex (male/female) were investigated in a separate multivariable binary logistic regression analysis and were not included as covariates in the histopathologic model.

Furthermore, singular R-CLA, singular R-FLU, and combined clarithromycin-fluoroquinolone resistance were analyzed. For each resistance type, a separate multivariable binary logistic regression analysis was performed. The respective resistance type was defined as the dependent variable (1 = respective resistance type, 0 = sensitive isolates and other resistance patterns). Here, too, all histopathologic parameters were simultaneously included in the model as independent variables, and the lowest category served as the reference.

As this was an exploratory analysis, no adjustment for multiple testing was performed. Results of small subgroups, particularly isolated R-FLU (n = 36), were interpreted with caution due to the small sample sizes and limited statistical power.

A p value of <0.05 was considered statistically significant. Analyses were carried out using Microsoft Excel (version 16.85; Microsoft Corporation, Redmond, WA) and the IBM Statistical Package for the Social Sciences (versions 27 and 29; IBM Corp., Armonk, NY).

Ethical statement

The ethics review committee of the Medical Association of Rhineland-Palatinate approved this study (Ref. 2023-16961). Moreover, the analyses were conducted in accordance with the Declaration of Helsinki, and all data were analyzed anonymously.

## Results

The cohort consisted of 374 patients, all of them with *H. pylori* PCR-positive FFPE samples (mean age, 53.4 years). Specifically, 59.9% of patients were female (n = 224) and 40.1% of patients were male (n = 150). Overall, resistance-associated mutations were detected in 251/374 patients (67.1%; male: n = 95, 37.8%; female: n = 156, 62.2%). Most resistant cases showed R-CLA (n = 163; 64.9%), followed by R-CLAxFLU (n = 52; 20.7%) and R-FLU (n = 36; 14.3%). The mean age of patients with resistance-associated mutations was 55.0 years (male: 53.1 years; female: 56.1 years).

In 123/374 patients (32.9%), no resistance-conferring mutations were found. In this susceptible category, the majority were female (n = 68; 55.3%), with a mean age of 48.8 years, whereas male patients (n = 55; 44.7%) had a mean age of 51.6 years. The mean age of the total cohort of susceptible, nonresistant patients was 50.1 years. The age difference between resistant and susceptible patients was statistically significant (p = 0.009), whereas sex was not significantly associated with resistance (p = 0.271; Table 1).

**Table 1:** Binary logistic regression analysis of the total cohort of H. pylori antibiotic-resistant patients associated with sex and age Hosmer-Lemeshow test: χ² = 5.277, df = 8, p = 0.728 CI: confidence interval; NA: not applicable; OR: odds ratio; REF: reference group

Parameter	OR (95% CI)	Wald χ²	p value
REF (≤20, male sex)	1.0	NA	NA
21-30 years	8.46 (1.84-38.88)	7.530	0.006
31-40 years	6.45 (1.62-25.60)	7.017	0.008
41-50 years	18.71 (4.46-78.48)	16.039	<0.001
51-60 years	11.16 (2.92-42.57)	12.464	<0.001
61-70 years	6.73 (1.79-25.39)	7.928	0.005
>70 years	14.50 (3.53-59.53)	13.767	<0.001
Age continuously	1.017 (1.004-1.03)	6.829	0.009
Female sex	1.29 (0.82-2.04)	1.210	0.271

Histologically, 213 patients (57.0%) exhibited mild gastritis activity, 118 (31.6%) showed moderate activity, and 20 (5.3%) showed severe activity; in 23 patients (6.1%), inflammatory activity was absent. Moreover, 87 patients (23.3%) had mild chronic gastritis, while 224 (59.9%) and 63 (16.8%) showed moderate and severe chronic gastritis, respectively. Based on *H. pylori* density, 168 patients (44.9%) had mild colonization, 149 (39.8%) had moderate colonization, and 57 (15.2%) had severe colonization. Atrophy was recorded in 48 patients (12.8%), and intestinal metaplasia in 54 patients (14.4%). Most patients showed either mild atrophy (46/48 cases) or mild intestinal metaplasia (49/54 cases). Owing to the low numbers of moderate-to-severe atrophy and intestinal metaplasia, the cohort groups “atrophy” and “intestinal metaplasia” were analyzed in total.

Logistic regression results concerning histological parameters are summarized in Tables 2, 3. Regarding the total cohort of *H. pylori*-resistant patients (Table 2), severe colonization (Hp +++) was the only statistically significant factor correlated to increased risk of *H. pylori*-resistance (p = 0.045). High chronic inflammation showed a trend as a potential factor but did not reach statistical significance (p = 0.060). With regard to the specific resistance subgroups (R-CLA, R-FLU, R-CLAxFLU), none of the examined histological parameters showed a statistically significant association. Atrophy showed only a nonsignificant trend in the R-FLU and R-CLA groups (p = 0.063 and p = 0.074, respectively; see Table 3).

**Table 2:** Binary logistic regression analysis of the total cohort of H. pylori antibiotic-resistant patients associated with the histological parameters according to the updated Sydney system Hosmer-Lemeshow test: χ² = 10.250, df = 8, p = 0.248 CI: confidence interval; NA: not applicable; OR: odds ratio; REF: reference group

Parameter	OR (95% CI)	Wald χ²	p value
Inflammatory activity			
No (REF)	1.0	NA	NA
Low	1.43 (0.57-3.58)	0.594	0.441
Moderate	1.60 (0.59-4.40)	0.844	0.358
High	0.83 (0.20-3.43)	0.064	0.800
Chronic inflammation			
Low (REF)	1.0	NA	NA
Moderate	0.60 (0.33-1.10)	2.779	0.096
High	0.45 (0.20-1.035)	3.532	0.060
Hp colonization			
+ (REF)	1.0	NA	NA
++	0.83 (0.51-1.36)	0.560	0.454
+++	2.26 (1.02-5.01)	4.029	0.045
Atrophy	1.39 (0.67-2.87)	0.774	0.379
Intestinal metaplasia	1.24 (0.62-2.48)	0.371	0.543

**Table 3:** Binary logistic regression analysis of R-CLA, R-FLU, and R-CLAxFLU with several histological parameters R-CLA: Hosmer-Lemeshow test: χ² = 2.619, df = 8, p = 0.956 R-FLU: Hosmer-Lemeshow test: χ² = 1.743, df = 8, p = 0.988 R-CLAxFLU: Hosmer-Lemeshow test: χ² = 3.981, df = 8, p = 0.859 CI: confidence interval; NA: not applicable; OR: odds ratio; REF: reference group; R-CLA: resistance to clarithromycin; R-FLU: resistance to fluoroquinolones; R-CLAxFLU: dual resistance

Parameter	R-CLA			R-FLU			R-CLAxFLU		
	OR (95% CI)	Wald χ²	p value	OR (95% CI)	Wald χ²	p value	OR (95% CI)	Wald χ²	p value
Inflammatory activity									
No (REF)	1.0	NA	NA	1.0	NA	NA	1.0	NA	NA
Low	0.67 (0.28-1.61)	0.813	0.367	2.77 (0.35-22.04)	0.922	0.337	4.00 (0.51-31.27)	1.742	0.187
Moderate	0.92 (0.35-2.43)	0.027	0.869	1.44 (0.16-13.22)	0.102	0.750	4.06 (0.48-34.44)	1.645	0.200
High	0.54 (0.14-2.12)	0.773	0.379	2.80 (0.19-42.35)	0.551	0.458	2.48 (0.18-34.48)	0.457	0.499
Chronic inflammation									
Low (REF)	1.0	NA	NA	1.0	NA	NA	1.0	NA	NA
Moderate	0.62 (0.35-1.09)	2.809	0.094	1.18 (0.48-2.90)	0.123	0.726	0.93 (0.42-2.08)	0.030	0.863
High	0.64 (0.30-1.41)	1.231	0.267	0.78 (0.20-3.08)	0.125	0.723	0.64 (0.20-2.06)	0.564	0.453
Hp colonization									
+ (REF)	1.0	NA	NA	1.0	NA	NA	1.0	NA	NA
++	0.85 (0.53-1.37)	0.468	0.494	1.47 (0.67-3.23)	0.909	0.340	0.73 (0.37-1.47)	0.767	0.381
+++	1.67 (0.85-3.29)	2.226	0.136	1.10 (0.33-3.63)	0.024	0.878	1.19 (0.48-2.92)	0.136	0.712
Atrophy	1.82 (0.94-3.52)	3.181	0.074	0.14 (0.02-1.11)	3.453	0.063	1.16 (0.47-2.83)	0.102	0.749
Intestinal metaplasia	0.91 (0.49-1.71)	0.087	0.768	1.65 (0.62-4.40)	1.004	0.316	1.22 (0.53-2.83)	0.221	0.638

## Discussion

In this study, we analyzed FFPE gastric biopsies with *H. pylori* gastritis to investigate the relationship between histopathological parameters and mutational status. The primary goal was to determine whether specific gastritis histopathological parameters were associated with *H. pylori* resistance. In particular, we first compared histopathology and overall resistance, followed by a specific comparison of R-CLA, R-FLU, and R-CLAxFLU. In a first step, we conducted a statistical analysis of our clinical data, especially regarding sex and age.

In a recently published large study cohort of 968 patients, we demonstrated that molecular testing of antibiotic resistance in *H. pylori* gastritis performed in FFPE specimens yields resistance patterns comparable to phenotypic analysis as the reference standard [24]. Consistent with previous data [24], the present results show that patients with resistant *H. pylori* infections were significantly older than those with susceptible infections. In addition, a relevant number of patients exhibited dual resistance to clarithromycin and fluoroquinolones, exceeding the number of isolated R-FLU cases. In contrast to our previous study, sex was not significantly associated with resistance in the present cohort. Heterogeneous results have also been reported by others, and even in our previous study, sex-dependent differences did not consistently occur across all subgroups.

The updated literature further emphasizes that antibiotic resistance in *H. pylori* is a rapidly evolving global clinical problem. Schulz et al. recently highlighted the urgent need for revised management strategies, improved surveillance, susceptibility-guided therapy, and novel treatment approaches in response to the worldwide increase in resistance [11]. Denis et al. also provided contemporary evidence supporting systematic susceptibility testing before first-line therapy, reinforcing the clinical relevance of early resistance-guided treatment [15].

In the past, several authors studied possible histological risk factors for the occurrence of *H. pylori* antibiotic resistance [21,22,27,28]. Among the histological parameters investigated, atrophy of gastric mucosa was particularly shown as a relevant factor. Goni et al. [21] demonstrated increasing R-CLA and metronidazole resistance with increasing severity of atrophy according to OLGA stage. Shao et al. [27] reported in a retrospective analysis of 2,847 patients that the rate of antibiotic resistance (measured by phenotypical analysis) in patients with atrophic gastritis was generally higher than that in other groups, indicating that patients with atrophic gastritis have a higher risk of infection with resistant strains. Xie et al. [28] also found higher rates of R-CLA and R-FLU in patients with chronic atrophic gastritis than in those with nonatrophic gastritis. Regarding FFPE specimens, Yüzügüldü et al. [22] examined gastric biopsy samples using microscopy and real-time PCR for R-CLA and identified atrophy as a significant parameter. In our FFPE-based morphomolecular analysis, gastric mucosal atrophy showed only a trend, not statistical significance, in its correlation with R-CLA or R-FLU. This difference may be explained by genetic variation, environmental factors, antibiotic exposure, and geographic heterogeneity between study populations [6,11].

In our study, strong *H. pylori* colonization (Hp +++ according to the updated Sydney system) was the only significant predictor in the total group of patients with *H. pylori* antibiotic resistance (p = 0.045). Interestingly, a significant correlation between the density and depth of *H. pylori* colonization and the severity of chronic gastritis in both overall and treatment-naïve patients has been described by Peng et al. [29]. From a pathophysiological perspective, increased *H. pylori* density may contribute to antibiotic resistance through biofilm-associated bacterial persistence. Biofilm formation may improve bacterial survival by reducing the effects of immune responses and antibiotics, thereby enhancing inflammatory reaction and tissue damage [29,30]. However, causality cannot be established in this retrospective study.

The association between strong colonization and resistance in the overall resistant cohort, but not in individual resistance subgroups, suggests that high bacterial density may represent a general marker of bacterial persistence rather than a specific predictor of resistance to a single antimicrobial class. From a practical perspective, *H. pylori* density may possibly serve as a pretreatment indicator of eradication effectiveness, as it may be associated with molecularly detected resistance. However, this hypothesis warrants further validation in prospective studies with phenotypic susceptibility testing and clinical eradication outcomes. In patients with high *H. pylori* density, quadruple eradication regimens containing low-resistance antibiotics may be especially relevant [27,31].

Including our previous assessment of FFPE gastric biopsies [24], the present data support morphomolecular analysis as a feasible method for simultaneous histological evaluation and routine *H. pylori* resistance testing. This approach provides actionable resistance information using routinely processed tissue with low failure rates. Even though several potential predictors of *H. pylori* resistance have been described, including increasing age, female sex, atrophy, and *H. pylori* density, published data remain heterogeneous [11,21,24,27]. Therefore, we support current recommendations favoring early resistance testing, including molecular analysis for mutations associated with R-CLA, R-FLU, resistance to tetracycline, and resistance to rifampicin [5,11,16].

This study has some limitations. First, it represents a monocentric, retrospective investigation of a routinely diagnosed patient cohort. Therefore, selection bias cannot be excluded. Second, patients with diagnosed gastritis referred for molecular analysis could differ from population-based cohorts, for example, with respect to former antibiotic treatment or eradication failure. Third, data on prior antibiotic treatment and eradication anamnesis were both not available and could not be implemented in the study. Fourth, phenotypic susceptibility testing and treatment outcome data were not available for all patients; therefore, direct correlation between molecular resistance findings and eradication success was not possible. Moreover, relying on molecularly predicted resistance instead of confirmed phenotypic susceptibility creates diagnostic and clinical risks [32]. While molecular assays quickly identify genetic markers (like specific genes or mutations), they cannot determine the exact functional expression of resistance or the actual minimum inhibitory concentration, as already stated by He et al. [32]. Nevertheless, this study evaluates, for the first time in a sizeable FFPE-based cohort, the relationship between specifically defined molecular resistance groups and histopathological findings according to the updated Sydney system.

## Conclusions

In this retrospective monocentric analysis of a cohort of 374 patients with chronic gastritis, we studied the relevant histological parameters according to the updated Sydney system as potential predictors for the occurrence of antibiotic resistance to *H. pylori,* including R-CLA, R-FLU, and R-CLAxFLU. High *H. pylori* density was the only statistically significant histopathological parameter associated with antibiotic resistance overall. Gastric mucosal atrophy, previously described as a relevant indicator, showed only a nonsignificant trend for R-FLU and R-CLA in our cohort. Even though several potential predictors for the increased risk of *H. pylori* antibiotic resistance are now known (such as increasing age, female sex, and microbial density, as shown in our study), there are still heterogeneous or even contradictory data, mostly due to geographic heterogeneities. Given increasing resistance rates worldwide, early molecular resistance testing is clinically important and may support individualized eradication therapy and improved antimicrobial stewardship. FFPE-based morphomolecular testing provides a practical diagnostic approach for routine pathology and may be particularly useful in centers without immediate access to culture-based susceptibility testing.
